# Impact of Drain Placement on Postoperative Complications after Thyroidectomy for Substernal Goiter

**DOI:** 10.1055/s-0043-1777804

**Published:** 2024-03-15

**Authors:** Usama Waqar, Ayesha Nasir Hameed, Meher Angez, Sudhesh Kumar, Hajra Arshad, Marium Tariq Siddiqui, Hira Khan, Werdah Viquar, Aiza Abbas, Arsalan Javid, Haissan Iftikhar, Syed Akbar Abbas, Huma Naz, Sarah Saleem

**Affiliations:** 1 Medical College, Aga Khan University, Karachi, Pakistan; 2 Department of Surgery, University Hospitals Birmingham, United Kingdom; 3 Department of Surgery, Section of Otolaryngology, Head and Neck Surgery, Aga Khan University Hospital, Karachi, Pakistan; 4 Gastroenterology and Surgery Service Line, Aga Khan University Hospital, Karachi, Pakistan; 5 Department of Community Health Sciences, Medical College, Aga Khan University, Karachi, Pakistan

**Keywords:** drainage, hematoma, substernal goiter, thyroid, thyroid surgery

## Abstract

**Introduction** Despite the evidence against drain placement after thyroidectomy, there is a lack of consensus on drain use in patients with substernal goiter.

**Objective** To assess the factors that increase the likelihood of drain placement and its impact on postoperative hematoma and other 30-day complications among adult patients undergoing thyroidectomy for substernal goiter.

**Methods** A retrospective cohort study that used data from the American College of Surgeons National Surgical Quality Improvement Program (ACS-NSQIP). Adult patients (aged ≥ 18 years) who underwent elective thyroidectomy for substernal goiter from 2016 to 2020 were included. Cases with closed suction neck drains placed upon completion of surgery were included in the drain group, and the remaining cases formed the nondrain group.

**Results** A total of 1,229 patients were included (46.5% with drain placement). The factors that increased the likelihood of drain placement included body mass index (BMI) ≥ 30 kg/m ^2^ , score between 3 and 5 on the American Society of Anesthesiologists (ASA) physical status classification, sternal split/transthoracic surgical approach, operative time ≥ 90 minutes, and surgery conducted by otolaryngologists. Patients with clean-contaminated or contaminated wound classifications were less likely to be submitted to drain placement. In addition, drain use had no impact on postoperative hematoma formation but was found to independently increase the risk of prolonged length of hospital stay.

**Conclusion** Thyroidectomy without drain placement might be safe for substernal goiter. However, this decision should be individualized for each patient.

Level Of Evidence: 3

## Introduction

Postoperative hematoma is among the most clinically significant complications following total thyroidectomy, particularly in patients with substernal goiter. 1 2 In cases of substernal goiter, there is close contact between the thyroid gland and vital aerodigestive and neurovascular structures. This poses several challenges for operating surgeons, including the extremely high risk of hematoma, among other complications. 3 4 A substernal goiter occupies a large space in the neck and in the superior mediastinum, which requires greater dissection of surrounding structures and longer duration of surgery.

Drain placement following thyroidectomy has been a standard practice in many surgical departments worldwide. 5 However, this has been declining as evidence has shown no significant impact of drain placement on the development of postoperative neck hematoma. 5 6 7 8 9 10 11 12 13 Contrarily, drain use has been associated with higher risks of other adverse outcomes. 5 7 14 Two recent meta-analyses 5 14 based on randomized controlled trials found that drain placement independently increased the likelihood of higher postoperative pain, prolonged hospital stay, and wound infections.

Despite the evidence against drain placement after thyroidectomy, 5 6 7 8 9 10 11 12 13 14 there is a lack of consensus on drain use in patients with substernal goiter. This could be attributed to many comparative studies excluding cases with substernal goiter, as identified by a recent systematic review. 14 In addition, substernal goiter has a low incidence, which makes it difficult to conduct adequately powered analyses. Based on the higher risk of hematoma in this cohort, Herranz and Latorre 15 advocate drain use despite a paucity of evidence comparing hematoma development among patients with and without drain placement.

In the present study, we assessed the factors that increase the likelihood of drain placement in patients undergoing thyroidectomy for substernal goiter using a multi-institutional database. We further explored the impact of drain placement on postoperative hematoma and other complications in this cohort.

## Methods

### Data Source, Study Design, and Population

The present was a multicenter, retrospective cohort study based on data from the American College of Surgeons National Surgical Quality Improvement Program (ACS-NSQIP). The ACS-NSQIP is a multi-institutional, nationally-validated database that evaluates surgical outcomes, with more than 700 partnering hospitals. Data are collected prospectively by trained surgical clinical reviewers (SCRs) at each hospital using a standardized protocol. The details on the sampling technique, data collection methodology, and measures of the ACS-NSQIP are described elsewhere. 16

The Strengthening the Reporting of Observational Studies in Epidemiology (STROBE) guidelines were followed in the present study, which was approved with exemption from consent by the Ethics Review Committee at Aga Khan University, Karachi, Pakistan (reference ID: 2022-7219-20725).

Adult patients (aged ≥18 years) who underwent elective thyroidectomy for substernal goiter from 2016 to 2020 were included. These cases were identified using the following current procedural terminology (CPT) codes: 60270 (thyroidectomy, including substernal thyroid; sternal split or transthoracic approach) and 60271 (thyroidectomy, including substernal thyroid; cervical approach). Cases with missing data in the ACS-NSQIP Thyroidectomy Targeted Dataset and those undergoing emergency or urgent surgeries were excluded. Since thyroidectomy is mostly performed by otolaryngologists or general surgeons, procedures performed by other principal surgical specialties were further excluded.

### Measures

The perioperative variables for risk adjustment included sociodemographic characteristics, medical comorbidities, and operative details. Sociodemographic characteristics comprised age, gender, race, Hispanic ethnicity, and body mass index (BMI). Race was categorized as white, black or African American, and others (which included Asians, Native Americans, Alaska natives, native Hawaiians, and Pacific islanders).

Comorbidities comprised diabetes mellitus, smoking, hypertension, bleeding disorder, previous neck surgery, and the score on the American Society of Anesthesiologists (ASA) physical status classification. Smoking status was quantified as smoking within one year of surgery. Bleeding disorders included hemophilia, vitamin K deficiency, thrombocytopenia, chronic anticoagulation therapy, and similar disorders predisposing patients to severe bleeding.

The operative characteristics comprised surgical approach, indication, principal specialty, central neck dissection, use of vessel sealant device, wound classification, and operative time. Surgical indications were categorized as benign or malignant based on the codes of the International Classification of Diseases, Ninth Revision, Clinical Modification (ICD-9-CM) and Tenth Revision (ICD-10-CM).

### Drain and Non-Drain Groups

Patients were categorized into drain and non-drain groups. Cases with closed suction neck drains placed upon completion of surgery were included in the drain group. The remaining cases formed the nondrain group.

### Outcomes

The primary outcome included 30-day postoperative neck hematoma. The ACS-NSQIP defines it as development of a hematoma or postoperative bleeding at the neck site. Ecchymosis or bruising alone does not qualify as hematoma. 17 Interventions required for hematoma management were also documented.

The secondary outcomes included any complication within 30 days, major morbidity, and prolonged length of stay. The complications were further divided into infectious and noninfectious. Infectious complications comprised surgical site infection (SSI), urinary tract infection (UTI), sepsis, septic shock, wound disruption, pneumonia, and clostridium difficile colitis. Noninfectious complications included cerebrovascular accident (CVA)/stroke, cardiac complications (myocardial infarction [MI] or cardiac arrest), pulmonary complications (ventilator dependence for > 48 hours or unplanned reintubation), acute renal failure, deep vein thrombosis (DVT), intra-/postoperative blood transfusion(s), postoperative hypocalcemia, and recurrent laryngeal nerve injury. Major morbidity included any of the following adverse outcomes: deep or organ space SSI, wound dehiscence, pulmonary embolism (PE), prolonged ventilation, unplanned reintubation, sepsis, septic shock, MI, cardiac arrest, or CVA. Prolonged length of hospital stay was defined as stay > 2 days during the index admission.

### Statistical Analysis

Descriptive statistics were reported. The Shapiro-Wilk test was used to confirm the nonparametric distribution of continuous variables, including age, BMI, operative time, and length of stay ( *p* < 0.001 for all). Accordingly, the continuous variables were reported using median and interquartile range (IQR) values and compared across the drain and non-drain groups using the Mann-Whitney U test. The categorical variables were expressed as frequencies and percentages, and the Chi-squared (χ ^2^ ) test or the Fisher exact test were computed to compare their differences across the drain and non-drain groups. Missing data were reported in tables to keep the denominators consistent in the calculations.

Multivariable binary logistic regression models were computed to explore independent factors increasing the likelihood of drain placement and to assess the impact of drain placement on postoperative neck hematoma. To select covariates for adjustment, variance inflation factors (VIFs) were calculated for clinically-relevant baseline characteristics, comorbidities, and operative variables with *p* < 0.20 on the univariate analysis. The threshold for significant multicollinearity was set at VIF ≥10, and no significant multicollinearity was observed during the analysis.

Separate binary logistic regression models were also computed to assess the impact of drain placement on any 30-day postoperative complication, major morbidity, and prolonged length of hospital stay. After ruling out significant multicollinearity, these models were adjusted for age, gender, BMI, ASA score, surgical approach, principal specialty, use of vessel sealants, wound classification, and operative time.

Two-sided analyses were conducted. The threshold for statistical significance was set at *p* < 0.05. Adjusted odds ratios (AORs) and their corresponding 95% confidence intervals (95%CIs) were reported. IBM SPSS Statistics for Windows (IBM Corp., Armonk, NY, United States), software, version 26.0, was used to conduct the analyses.

## Results

### Patient Characteristics

**Fig. 1:**
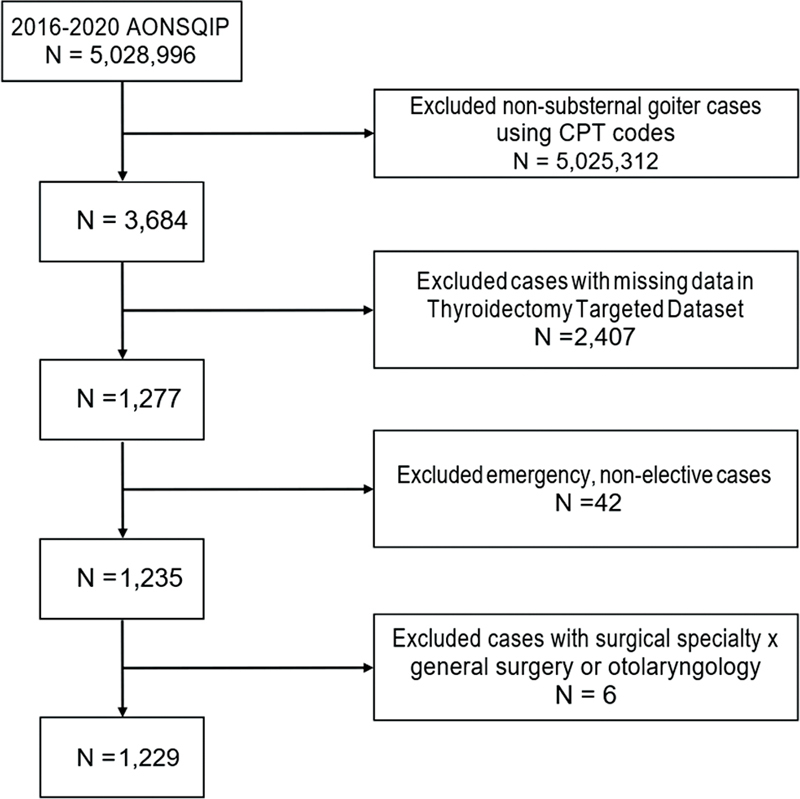
Details of the total cases included and the cases that were excluded based on CPT codes, missing data, emergency/ non-elective procedures, and ineligible surgeon subspecialties.

A total of 1,229 participants were included in the present study ( Fig. 1 ). Most patients were female (72.2%), had a median age of 59.0 ([IQR 22.0) years, and were operated on for benign indications (89.2%). Other baseline characteristics, comorbidities, and operative details are summarized in Table 1 .

**Table 1:** Baseline characteristics, comorbidities, and operative variables stratified by drain status

Variable	No drain *n* = 657	Drain *n* = 572	*p-value*
Age in years*	59.0 (22.0)	59.0 (22.0)	0.278
* < 65* * ≥ 65*	440 (67.0%) 217 (33.0%)	368 (64.3%) 204 (35.7%)	0.332
**Gender** * Female* * Male*	498 (75.8%) 159 (24.2%)	389 (68.0%) 183 (32.0%)	0.002**
**Race** * White* * Black or African American* * Others* * Missing*	337 (56.4%) 242 (40.5%) 18 (3.0%) 60	286 (57.2%) 197 (39.4%) 17 (3.4%) 72	0.886
**Hispanic ethnicity** * Missing*	39 (6.4%) 50	32 (6.2%) 55	0.872
BMI (kg/m ^2^ )*	31.8 (9.6)	34.3 (11.4)	< 0.001**
BMI (kg/m ^2^ * < 25.0* * 25.0–30.0* * ≥ 30.0* * Missing*	96 (14.7%) 155 (23.7%) 403 (61.6%) 3	54 (9.6%) 112 (19.8%) 399 (70.6%) 7	0.002**
**ASA score** * 1–2* * 3–5* * Missing*	304 (46.3%) 352 (53.7%) 1	196 (34.4%) 374 (65.6%) 2	< 0.001**
**Diabetes mellitus**	121 (18.4%)	127 (22.2%)	0.099
**Current smoker**	88 (13.4%)	90 (15.7%)	0.245
**Hypertension**	351 (53.4%)	323 (56.5%)	0.285
**Bleeding disorder**	8 (1.2%)	17 (3.0%)	0.030**
**Previous neck surgery**	53 (8.1%)	48 (8.4%)	0.836
**Surgical approach** * Cervical* * Sternal split or transthoracic*	587 (89.3%) 70 (10.7%)	488 (85.3%) 84 (14.7%)	0.033**
**Indication** * Benign* * Malignant* * Missing*	591 (90.1%) 65 (9.9%) 1	505 (88.3%) 67 (11.7%) 0	0.308
**Specialty** * General surgery* * Otolaryngology*	466 (70.9%) 191 (29.1%)	190 (33.2%) 382 (66.8%)	< 0.001**
**Central neck dissection** * Missing*	70 (10.8%) 9	66 (11.6%) 4	0.652
**Vessel sealant device** * Missing*	508 (78.8%); 12	379 (68.2%); 16	< 0.001**
**Wound classification** * Clean* * Clean-contaminated or contaminated*	624 (95.0%); 33 (5.0%)	564 (98.6%); 8 (1.4%)	< 0.001**
Operative time in minutes*	114.0 (73.0)	150.0 (100.0)	< 0.001**
Operative time/mins * < 90* * 90–120* * 120–150* * > 150*	211 (32.1%) 140 (21.3%) 125 (19.0%) 181 (27.5%)	80 (14.0%) 109 (19.1%) 95 (16.6%) 288 (50.3%)	< 0.001**

Abbreviations: ASA, American Society of Anesthesiologists; BMI, body mass index.

Notes: *Reported as median and interquartile range values; **statistically significant ( *p* < 0.05); percentages are presented in columns.

Drains were placed in 46.5% of the cases. Compared with the non-drain group, a greater proportion of patients in the drain group: were male (32.0% versus 24.2%), had a BMI ≥ 30 kg/m ^2^ (70.6% versus 61.6%), presented ASA scores from 3 to 5 (65.6% versus 53.7%), presented bleeding disorders (3.0 versus 1.2%), had operative time ≥ 150 minutes (50.3% versus 27.5%), and underwent a sternal split or transthoracic approach (14.7% versus 10.7%). Similarly, having a clean wound classification was significantly more common among patients in the drain group compared to those in the non-drain group (98.6% versus 95.0%). Additionally, otolaryngologists were more likely to use drains (66.8%) in comparison with general surgeons (33.2%). Use of vessel sealant devices was more common in the non-drain group (78.8%) than in the drain group (68.2%).

### Factors Increasing the Likelihood of Drain Placement

After adjustment for baseline characteristics, comorbidities, and operative details, factors increasing the likelihood of drain placement included BMI ≥ 30 kg/m ^2^ , ASA score from 3 to 5, sternal split/transthoracic approach, operative time ≥ 90 minutes, and surgery conducted by otolaryngologists ( Table 2 ). Patients with clean-contaminated or contaminated wound classifications were less likely to undergo drain placement (AOR: 0.214; 95%CI: 0.089–0.514).

**Table 2:** Univariate and multivariable binary logistic regression for drain placement among patients undergoing thyroidectomy for substernal goiter

Variable	Unadjusted odds ratio	*p-value*	Adjusted odds ratio	*p-value*
**Age in years** * < 65* * ≥ 65*	Reference 1.124 [0.888–1.423]	0.332	**-**	**-**
**Gender** * Female* * Male*	Reference 1.473 [1.147–1.893]	0.002**	Reference 1.230 [0.910–1.663]	0.177
**Race** * White* * Black or African American* * Others*	Reference 0.959 [0.751–1.226] 1.113 [0.563–2.200]	0.739 0.758	-	-
**Hispanic ethnicity**	0.961 [0.593–1.558]	0.872	−	−
** BMI (kg/m ^2^ ) ** * < 25.0* * 25.0–30.0* * ≥ 30.0*	Reference 1.285 [0.850–1.941] 1.760 [1.227–2.525]	0.234 0.002**	Reference 1.287 [0.786–2.108] 1.675 [1.078–2.602]	0.316 0.022*
**ASA score** * 1–2* * 3–5*	Reference 1.648 [1.308–2.076]	< 0.001**	Reference 1.415 [1.060–1.887]	0.018*
**Diabetes mellitus**	1.264 [0.957–1.671]	0.099	1.240 [0.876–1.757]	0.225
**Current smoker**	1.207 [0.879–1.659]	0.245	−	−
**Hypertension**	1.131 [0.903–1.417]	0.285	−	−
**Bleeding disorder**	2.485 [1.064–5.802]	0.035*	2.321 [0.863–6.245]	0.095
**Previous neck surgery**	1.044 [0.694–1.569]	0.836	−	−
**Surgical approach** * Cervical* * Sternal split or transthoracic*	Reference 1.443 [1.028–2.026]	0.034*	Reference 1.756 [1.167–2.640]	0.007*
**Indication** * Benign* * Malignant*	Reference 1.206 [0.841–1.731]	0.309	-	-
**Specialty** * General surgery* * Otolaryngology*	Reference 4.905 [3.850–6.249]	< 0.001**	Reference 6.932 [5.179–9.278]	< 0.001**
**Central neck dissection**	1.086 [0.760–1.551]	0.652	−	−
**Vessel sealant device**	0.557 [0.445–0.749]	< 0.001**	0.803 [0.587–1.099]	0.171
**Wound classification** * Clean* * Clean-contaminated or contaminated*	Reference 0.268 [0.123–0.586]	0.001**	Reference 0.214 [0.089–0.514]	0.001**
**Operative time in minutes** * < 90* * 90* – *120* * 120* – *150* * > 150*	Reference 2.053 [1.434–2.940] 2.004 [1.384–2.904] 4.197 [3.055–5.765]	< 0.001** < 0.001** < 0.001**	Reference 2.495 [1.651–3.772] 2.637 [1.714–4.057] 6.323 [4.323–9.248]	< 0.001** < 0.001** < 0.001**

Abbreviations: ASA, American Society of Anesthesiologists; BMI, body mass index.

Notes: **Statistically significant ( *p* < 0.05); only cases with complete data on all covariates were included ( *n* = 1,189).

### Postoperative Hematoma

A total of 2.2% of the patients (27 of 1,229) developed postoperative hematoma. Most hematoma cases were underwent additional observation during hospital admission (70.4%; 19 of 27) while 1 patient required tracheostomy (3.7%; 1 of 27). The remaining seven patients did not require additional observation.

On the univariate analysis, there was no significant difference in the incidence of neck hematoma in the drain and non-drain groups (3.0% versus 1.5% respectively; *p* = 0.083). This was consistent across the multivariable regression analysis after adjustment for clinically relevant characteristics (AOR: 1.289; 95%CI: 0.525–3.168). Instead, black or African American race was identified as a risk factor for postoperative hematoma, while use of vessel sealant devices was found to independently reduce the risk of hematoma development ( Table 3 ).

**Table 3:** Univariate and multivariable binary logistic regression analyses for postoperative hematoma

Variable	Unadjusted odds ratio	*p* -value	Adjusted odds ratio	*p* -value
**Age in years** * < 65* * ≥ 65*	Reference 1.336 [0.614–2.906]	0.465	-	-
** Gender**				
* Female* * Male*	Reference 1.304 [0.580–2.932]	0.520	−	−
** Race** * White* * Black or African American* * Others*	Reference 2.536 [1.055–6.099] 2.254 [0.274–18.540]	0.038** 0.450	Reference 3.239 [1.223–8.574] 2.142 [0.229–20.008]	0.018** 0.504
**Hispanic ethnicity**	2.787 [0.934–8.319]	0.066	3.572 [0.684–18.652]	0.131
** BMI (kg/m ^2^ ) ** * < 25.0* * 25.0* – *30.0* * ≥ 30.0*	Reference 0.738 [0.163–3.342] 1.238 [0.363–4.219]	0.693 0.733	−	−
** ASA score** * 1* – *2* * 3* – *5*	Reference 3.101 [1.166–8.244]	0.023**	Reference 2.480 [0.888–6.929]	0.083
**Diabetes mellitus**	1.681 [0.727–3.886]	0.225	−	−
**Current smoker**	1.716 [0.683–4.313]	0.251	−	−
**Hypertension**	1.664 [0.741–3.733]	0.217	−	−
**Bleeding disorder**	1.881 [0.245–14.437]	0.543	−	−
**Previous neck surgery**	1.996 [0.677–5.891]	0.210	−	−
** Surgical approach** * Cervical* * Sternal split or transthoracic*	Reference 0.551 [0.129–2.348]	0.420	−	−
** Indication** * Benign* * Malignant*	Reference 0.313 [0.042–2.325]	0.256	−	−
**Specialty** * General surgery* * Otolaryngology*	Reference 1.439 [0.668–3.099]	0.353	−	−
**Central neck dissection**	1.464 [0.497–4.315]	0.489	−	−
**Vessel sealant device**	0.249 [0.113–0.549]	0.001**	0.167 [0.070–0.401]	< 0.001**
**Wound classification** * Clean* * Clean-contaminated or contaminated*	Reference Could not be computed	−	−	−
** Operative time in minutes** * < 90;* * 90–120;* * 120–150;* * > 150*	Reference; 2.375 [0.706–7.984]; 1.319 [0.326–5.335]; 1.715 [0.541-5.437]	0.162 0.697 0.360	Reference; 2.141 [0.530–8.649]; 1.242 [0.264–5.836]; 0.906 [0.216–3.790]	0.285 0.784 0.892
**Drain placement**	1.983 [0.901–4.366]	0.089	1.289 [0.525–3.168]	0.580

Abbreviations: ASA, American Society of Anesthesiologists; BMI, body mass index.

Notes: **Statistically significant ( *p* < 0.05); only cases with complete data on all covariates were included ( *n* = 1,052).

### Complications

The univariate analyses demonstrated no significant differences in terms of major morbidity, infectious complications, sepsis, wound disruption, pneumonia, cardiac complications, acute renal failure, postoperative hypocalcemia, recurrent laryngeal nerve injury, unplanned readmission, and mortality between the both groups. However, compared with the non-drain group, patients with drain placement presented significantly higher occurrences of surgical site infections (0.7% versus 0.0%; *p* = 0.047), unplanned reintubation (1.6% versus 0.5%; *p* = 0.047), blood transfusions (1.9% versus 0.3%; *p* = 0.006), unplanned reoperations (3.0 versus 0.9%; *p* = 0.008), and prolonged length of hospital stay (15.9% versus 5.0%; *p* < 0.001) ( Table 4 ).

**Table 4:** Thirty-day postoperative complications, stratified by drain status

Variable	No drain *n* = 657	Drain *n* = 572	*p-value*
**Neck hematoma** * Missing*	10 (1.5%) 2	17 (3.0%) 2	0.083
**Any complication**	87 (13.2%)	78 (13.6%)	0.840
**Major morbidity**	11 (1.7%)	11 (1.9%)	0.743
**Infectious complications**	8 (1.2%)	7 (1.2%)	0.992
**Surgical site infection**	0 (0.0%)	4 (0.7%)	0.047**
**Urinary tract infection**	3 (0.5%)	0 (0.0%)	0.253
**Sepsis**	1 (0.2%)	0 (0.0%)	> 0.999
**Septic shock**	1 (0.2%)	0 (0.0%)	> 0.999
**Wound disruption**	2 (0.3%)	1 (0.2%)	> 0.999
**Pneumonia**	3 (0.5%)	1 (0.2%)	0.628
**Clostridium difficile colitis**	0 (0.0%)	1 (0.2%)	0.465
**Noninfectious complications**	83 (12.6%)	74 (12.9%)	0.874
**CVA/stroke with neurological deficit**	1 (0.2%)	0 (0.0%)	> 0.999
**Cardiac complications** * Myocardial infarction* * Cardiac arrest requiring CPR*	2 (0.3%) 2 (0.3%) 0 (0.0%)	2 (0.3%) 0 (0.0%) 2 (0.3%)	> 0.999 0.502 0.216
**Pulmonary complications** * Ventilator > 48 hours* * Unplanned intubation*	5 (0.8%) 3 (0.5%) 3 (0.5%)	11 (1.9%) 4 (0.7%) 9 (1.6%)	0.073 0.711 0.047**
**Acute renal failure**	1 (0.2%)	0 (0.0%)	> 0.999
**DVT/thrombophlebitis**	1 (0.2%)	0 (0.00%)	> 0.999
**Blood transfusion**	2 (0.3%)	11 (1.9%)	0.006**
**Postoperative hypocalcemia** * Missing*	31 (4.9%) 21	24 (4.4%) 28	0.707
**Recurrent laryngeal nerve injury** * Missing*	46 (7.0%) 3	39 (6.9%) 5	0.915
**Unplanned reoperation**	6 (0.9%)	17 (3.0%)	0.008**
**Unplanned readmission**	18 (2.7%)	17 (3.0%)	0.807
Length of stay*	1.0 (1.0)	1.0 (1.0)	< 0.001**
**Prolonged length of stay** * Missing*	33 (5.0%) 0	91 (15.9%) 1	< 0.001**
**Mortality**	2 (0.3%)	0 (0.0%)	0.502

Abbreviations: CPR, cardiopulmonary resuscitation; CVA, cerebrovascular accident; DVT, deep vein thrombosis.

Notes: *Reported as median and interquartile range values; **statistically significant ( *p* < 0.05); percentages are presented in columns.

The results of the regression analysis of the association of drain placement with postoperative outcomes are shown in Table 5 . After adjustment for clinically relevant covariates, drain use in patients with thyroidectomy for substernal goiter independently increased the likelihood of prolonged length of hospital stay (AOR: 2.047; 95%CI: 1.245–3.368). Drain placement was not independently associated with any postoperative complication or major morbidity ( Table 5 ).

**Table 5:** Multivariable binary logistic regression analyses for other postoperative outcomes with drain status as the main explanatory covariate

Outcome	Adjusted odds ratio	*p-value*
**Any complication**	0.698 [0.469-1.037]	0.075
**Major morbidity**	0.518 [0.183-1.467]	0.215
**Prolonged length of stay**	2.047 [1.245-3.368]	0.005**

Notes: No drain group was reference; regression adjusted for age, gender, body mass index (BMI), score on the American Society of Anesthesiology (ASA) physical status classification, surgical approach, principal specialty, use of vessel sealants, wound classification, and operative time; **statistically significant ( *p* < 0.05); only cases with complete data on all covariates were included ( *n* = 1,188 for prolonged length of stay and 1,189 for all other outcomes).

## Discussion

The present study used a large, multi-institutional dataset to compare the incidence of postoperative hematoma and other complications among patients undergoing thyroidectomy for substernal goiter with and without drain use. Our results demonstrate that drain use was associated with 28.9% higher odds of hematoma, but this was not statistically significant. We speculate that this may be due to a higher probability of detecting clinically-insignificant minor hematomas during hospital admission. This hypothesis is supported by the longer length of hospital stay observed among patients with drain compared with the non-drain cohort, which provided more time to detect insignificant hematomas. In contrast, an alternative explanation for the higher likelihood of hematoma formation could be the presence of inherent selection biases for drain placement in high-risk cases. Our analysis demonstrated that ASA scores from 3 to 5, BMI ≥ 30 kg/m ^2^ , and more complex surgical approaches were independently associated with drain use, further supporting this hypothesis. More appropriate variables to assess the risk of hematoma in this population would include size of resection, intraoperative hemorrhage, surgeon experience, and use of preoperative anticoagulation. 7 However, our regression analyses could not be adjusted for these variables, as they are not captured in the ACS-NSQIP, limiting the conclusiveness of our findings. Nevertheless, these results are in accordance with the existing literature on the impact of drain use on postoperative hematoma in thyroidectomies. 5 6 7 8 9 10 11 12 13

In the present study, drain use was independently associated with prolonged length of stay. This is consistent among studies on cases of non-substernal goiter undergoing thyroidectomy. 7 14 This could be attributed to surgeon reluctance to discharge patients until the drain output thresholds are met. While beyond the scope of the current study, prolonged length of stay can impose higher monetary costs on patients, lead to patient discomfort, and burden the limited hospital resources available. 18 19 In addition, prolonged hospital stay may predispose patients to nosocomial infections. Even though drain use did not independently increase the risk of developing infectious complications, the univariate analysis revealed a higher likelihood of developing infectious complications among patients with prolonged versus non-prolonged stay (4.0% versus 0.8% respectively; *p* = 0.009).

We found that otolaryngologists were more likely to use drains in thyroidectomy for substernal goiter compared with general surgeons (AOR: 6.932; 95%CI: 5.179–9.278), even after adjustment for relevant baseline, patient, and surgery characteristics. However, there was no significant difference in the rate of postoperative hematoma among their cases (1.8% in general surgery cases versus 2.6% in otolaryngology cases; *p* = 0.351). In addition, patients operated on by otolaryngologists were more likely to have prolonged hospital stay compared with general surgery patients (12.9% versus 7.6%, respectively; *p* = 0.002). Chung et al. 20 report similar findings in their comparison of thyroidectomies performed by general surgeons versus otolaryngologists. These apparent differences in length of hospital stay might be a surrogate measure for differences in drain placement practices among these two specialties.

Surgeons were less likely to place drains in clean-contaminated or contaminated wounds. Considering thyroidectomy is a clean procedure, this seems to have occurred in cases in which concomitant procedures such as tracheostomy were performed. We speculate that this would be when tracheomalacia or bilateral cord paralysis was suspected.

Several implications can be inferred from the present study. First, thyroidectomy without drain placement might be safe among patients with substernal goiter. However, this decision should be individualized for each patient, while also accounting for surgeon preference. Specifically considering the similar rates of postoperative hematoma observed among surgeries conducted by otolaryngologists and gender surgeons, otolaryngologists may better optimize their practice regarding patient selection for drain placement. The conclusiveness of these recommendations, however, is limited to patients without preoperative anticoagulation therapy or significant intraoperative bleeding, considering these factors could not be explored in the current study.

Second, a shorter hospital stay might be feasible with proper patient education related to drain management in certain cases in which drains are still indicated. 21 Familiarizing surgeons with short-stay protocols might also help avoid unnecessary hospital stays. However, this requires further evaluation, as patient education and short-stay protocols were beyond the scope of the current study.

The present study has multiple limitations which should be considered while interpreting its results. Since we used an existing database, our analyses are limited to variables included within the dataset. The ACS-NSQIP does not include the extent of hematoma, development of seroma, surgeon experience, and hospital volume. In addition, preoperative laboratory assessments could not be included in the statistical analyses because of the substantial number of missing values. These factors might be associated with hematoma formation in this cohort. Lastly, the database might be susceptible to coding and data collection errors.

## Conclusion

Thyroidectomy without drain placement might be safe for substernal goiter, particularly in patients without preoperative anticoagulation use or intraoperative hemorrhage. However, this decision should be individualized for each patient, while accounting for surgeon preference. Otolaryngologists may better optimize their patient selection practice for drain placement. In addition, patient education regarding drain management and short-stay protocols should be further evaluated to help reduce unnecessary hospital stays.

## References

[1] Fan C Zhou X Su G Risk factors for neck hematoma requiring surgical re-intervention after thyroidectomy: a systematic review and meta-analysis BMC Surg 2019 19 01 98 31340806 10.1186/s12893-019-0559-8 PMC6657038

[2] Doulaptsi M Karatzanis A Prokopakis E Substernal goiter: Treatment and challenges. Twenty-two years of experience in diagnosis and management of substernal goiters Auris Nasus Larynx 2019 46 02 246 251 30055961 10.1016/j.anl.2018.07.006

[3] Hanson M A Shaha A R Wu J X Surgical approach to the substernal goiter Best Pract Res Clin Endocrinol Metab 2019 33 04 101312 31477522 10.1016/j.beem.2019.101312 PMC6815725

[4] Farooq M S Nouraei R Kaddour H Saharay M Patterns, timing and consequences of post-thyroidectomy haemorrhage Ann R Coll Surg Engl 2017 99 01 60 62 27551897 10.1308/rcsann.2016.0270 PMC5392799

[5] Portinari M Carcoforo P The application of drains in thyroid surgery Gland Surg 2017 6 05 563 573 29142849 10.21037/gs.2017.07.04 PMC5676181

[6] Li L Chen H Tao H The effect of no drainage in patients who underwent thyroidectomy with neck dissection: A systematic review and meta-analysis Medicine (Baltimore) 2017 96 50 e9052 29390300 10.1097/MD.0000000000009052 PMC5815712

[7] Maroun C A El Asmar M Park S J Drain placement in thyroidectomy is associated with longer hospital stay without preventing hematoma Laryngoscope 2020 130 05 1349 1356 31508818 10.1002/lary.28269

[8] Abboud B El-Kheir A Redo thyroid surgery without drains Surg Today 2020 50 12 1619 1625 32623584 10.1007/s00595-020-02065-9

[9] Hua N Quimby A E Johnson-Obaseki S Comparing hematoma incidence between hemostatic devices in total thyroidectomy: a systematic review and meta-analysis Otolaryngol Head Neck Surg 2019 161 05 770 778 31331260 10.1177/0194599819865248

[10] Mekel M Stephen A E Gaz R D Surgical drains can be safely avoided in lateral neck dissections for papillary thyroid cancer Am J Surg 2010 199 04 485 490 20359568 10.1016/j.amjsurg.2009.04.006

[11] Al-Qahtani A S Abouzeid Osman T Could post-thyroidectomy bleeding be the clue to modify the concept of postoperative drainage? A prospective randomized controlled study Asian J Surg 2018 41 05 511 516 29037884 10.1016/j.asjsur.2017.08.004

[12] Abboud B Tannoury J Sleilaty G Daher R Abadjian G Ghorra C Cervical neck dissection without drainage in papillary thyroid carcinoma J Laryngol Otol 2013 127 03 299 302 23374592 10.1017/S0022215112003222

[13] Lee S W Choi E C Lee Y M Lee J Y Kim S C Koh Y W Is lack of placement of drains after thyroidectomy with central neck dissection safe? A prospective, randomized study Laryngoscope 2006 116 09 1632 1635 16954994 10.1097/01.mlg.0000231314.86486.be

[14] Soh T CF Ong Q J Yip H M Complications of Neck Drains in Thyroidectomies: A Systematic Review and Meta-Analysis Laryngoscope 2021 131 03 690 700 33022081 10.1002/lary.29077

[15] Herranz J Latorre J [Drainage in thyroid and parathyroid surgery] Acta Otorrinolaringol Esp 2007 58 01 7 9 17371671

[16] Principal Site Investigators of the Patient Safety in Surgery Study Khuri S F Henderson W G Daley J The patient safety in surgery study: background, study design, and patient populations J Am Coll Surg 2007 204 06 1089 1102 17544068 10.1016/j.jamcollsurg.2007.03.028

[17] American College of Surgeons National Surgical Quality Improvement Program. User Guide for the2020 ACS NSQIP Participant Use Data File (PUF) https://www.facs.org/-/media/files/quality-programs/nsqip/nsqip_puf_userguide_2020.ashx. Accessed March 10, 2022.

[18] Hurtado-López L M López-Romero S Rizzo-Fuentes C Zaldívar-Ramírez F R Cervantes-Sánchez C Selective use of drains in thyroid surgery Head Neck 2001 23 03 189 193 11428448 10.1002/1097-0347(200103)23:3<189::aid-hed1017>3.0.co;2-y

[19] Schoretsanitis G Melissas J Sanidas E Christodoulakis M Vlachonikolis J G Tsiftsis D D Does draining the neck affect morbidity following thyroid surgery? Am Surg 1998 64 08 778 780 9697913

[20] Chung P J Lee M Chang E H Does specialty matter? Analysis of outcomes in total thyroidectomy for goiters between general surgery and otolaryngology using American College of Surgeons NSQIP J Am Coll Surg 2017 225 04 S68

[21] Ha P K Couch M E Tufano R P Koch W M Califano J A Short hospital stay after neck dissection Otolaryngol Head Neck Surg 2005 133 05 677 680 16274791 10.1016/j.otohns.2005.07.029

